# Development and Validation of an Explainable Machine Learning Model for Predicting Myocardial Injury After Noncardiac Surgery in Two Centers in China: Retrospective Study

**DOI:** 10.2196/54872

**Published:** 2024-07-26

**Authors:** Chang Liu, Kai Zhang, Xiaodong Yang, Bingbing Meng, Jingsheng Lou, Yanhong Liu, Jiangbei Cao, Kexuan Liu, Weidong Mi, Hao Li

**Affiliations:** 1 Department of Anesthesiology, The First Medical Center, Chinese People's Liberation Army General Hospital, 28th Fuxing Road, Haidian District, Beijing, 100853, China, 86 15010665099; 2 Medical School of Chinese People's Liberation Army General Hospital, Beijing, China; 3 National Clinical Research Center for Geriatric Diseases, Chinese People's Liberation Army General Hospital, Beijing, China; 4 Institute of Computing Technology Chinese Academy of Science, Beijing, China; 5 Department of Anesthesiology, Nanfang Hospital, Southern Medical University, Guangzhou, China

**Keywords:** myocardial injury after noncardiac surgery, older patients, machine learning, personalized prediction, myocardial injury, risk prediction, noncardiac surgery

## Abstract

**Background:**

Myocardial injury after noncardiac surgery (MINS) is an easily overlooked complication but closely related to postoperative cardiovascular adverse outcomes; therefore, the early diagnosis and prediction are particularly important.

**Objective:**

We aimed to develop and validate an explainable machine learning (ML) model for predicting MINS among older patients undergoing noncardiac surgery.

**Methods:**

The retrospective cohort study included older patients who had noncardiac surgery from 1 northern center and 1 southern center in China. The data sets from center 1 were divided into a training set and an internal validation set. The data set from center 2 was used as an external validation set. Before modeling, the least absolute shrinkage and selection operator and recursive feature elimination methods were used to reduce dimensions of data and select key features from all variables. Prediction models were developed based on the extracted features using several ML algorithms, including category boosting, random forest, logistic regression, naïve Bayes, light gradient boosting machine, extreme gradient boosting, support vector machine, and decision tree. Prediction performance was assessed by the area under the receiver operating characteristic (AUROC) curve as the main evaluation metric to select the best algorithms. The model performance was verified by internal and external validation data sets with the best algorithm and compared to the Revised Cardiac Risk Index. The Shapley Additive Explanations (SHAP) method was applied to calculate values for each feature, representing the contribution to the predicted risk of complication, and generate personalized explanations.

**Results:**

A total of 19,463 eligible patients were included; among those, 12,464 patients in center 1 were included as the training set; 4754 patients in center 1 were included as the internal validation set; and 2245 in center 2 were included as the external validation set. The best-performing model for prediction was the CatBoost algorithm, achieving the highest AUROC of 0.805 (95% CI 0.778‐0.831) in the training set, validating with an AUROC of 0.780 in the internal validation set and 0.70 in external validation set. Additionally, CatBoost demonstrated superior performance compared to the Revised Cardiac Risk Index (AUROC 0.636*; P*<.001). The SHAP values indicated the ranking of the level of importance of each variable, with preoperative serum creatinine concentration, red blood cell distribution width, and age accounting for the top three. The results from the SHAP method can predict events with positive values or nonevents with negative values, providing an explicit explanation of individualized risk predictions.

**Conclusions:**

The ML models can provide a personalized and fairly accurate risk prediction of MINS, and the explainable perspective can help identify potentially modifiable sources of risk at the patient level.

## Introduction

Myocardial injury after noncardiac surgery (MINS), a prominent postoperative cardiovascular complication, occurs in approximately 8% to 22% of patients overall [1]. The Vascular Events in Noncardiac Surgery Patients Cohort Evaluation (VISION) study showed that MINS was the second most common cause of short-term mortality among 8 perioperative adverse events [2,3]. MINS is also reportedly an independent predictor of 1-year or long-term mortality [4]. Nevertheless, 90% of the MINS events are unrecognized because most patients are not presenting ischemic symptoms, and a minority of MINS cases are diagnosed by electrocardiogram abnormalities, involving typical chest pain symptoms [5]. Therefore, early prediction and identification of patients at higher risk for MINS is critically important for enhancing the outcomes of these underappreciated complications in older patients.

The most common prediction tool available to identification of high-risk patients is Revised Cardiac Risk Index (RCRI) [6], a universally used screening tool due to ease of use but with poor performance in other validation sets. American College of Surgeons National Surgeons Quality Improvement Program (NSQIP) [7] and Myocardial Infarction or Cardiac Arrest (MICA) surgical risk calculators [8] were subsequently developed with higher accuracy than RCRI, designed to predict more severe outcomes, including death and myocardial infarction, instead of predicting MINS. Another prediction model was derived from the MANAGE cohort [9], using 3 preoperative risk factors and not considering intraoperative factors. ML has been proven more powerful than conventional logistic regression because it can overcome the limitations of statistical methods and even create personalized risk predictions [10]. Recently, two novel ML models were reported to predict the occurrence of MINS. Oh et al [11] developed a machine learning (ML) model and achieved an area under the receiver operating characteristic (AUROC) curve of 0.78 using 12 variables. However, the population heterogeneity and lack of external validation may limit its generalization to older patients. Nolde et al [12] applied single-layer and multiple-layer variables to different models and achieved the highest AUROC of 0.77 and accuracy of 0.70. Despite comprehensive included variables, anesthesiologists and surgeons are unable to distinguish modifiable risk factors and make targeted interventions to improve outcomes.

Currently, no validated and accurate risk prediction tools for MINS are in use. Therefore, the purpose of our research was to develop and validate an ML model that predicts MINS risk based on surgery data available at admission and during the intraoperative period. The model also used Shapley Additive Explanations (SHAP) method to interpret results, allowing for targeted interventions to modify risk factors and support clinical decision-making.

## Methods

### Patient Cohort

We collected data anonymously from our electronic health record (EHR) system, which was an integrated clinical database containing data on all patients who were admitted to hospitals. The data set was derived from older patients (defined as aged ≥65 years) undergoing noncardiac surgery from January 2017 to August 2019, and the internal validation data set was derived from patients enrolled from July 2020 to July 2021 in center 1 (Chinese People's Liberation Army General Hospital in northern China). We also included patients who had noncardiac surgeries in center 2 (Nanfang Hospital of Southern Medical University in southern China) from January 2021 to October 2021 as an external data set. The uniform exclusion criteria were as follows: excluding patients with the American Society of Anesthesiologists (ASA) grade V, a short operation interval (scheduled for more than 1 surgery within a week), with nongeneral anesthesia, low-risk surgery (eg, outpatient surgery, hysteroscopic surgery, or body surface surgery), and a short surgery duration (≤30 min). Patients undergoing either elective or emergency surgery were eligible for participation.

### Ethical Considerations

The study was approved by the Ethics Committee Board of the First Medical Center of Chinese People's Liberation Army (S2019-311-02), and the requirement for informed consent was waived because this was an observational study with minimal risk for patients. This study conforms to the principles outlined in the Transparent Reporting of a multivariable prediction model for Individual Prognosis or Diagnosis (TRIPOD) statement.

### Data Processing

Variables from the following categories were collected: demographics, preoperative comorbid conditions and medications, preoperative laboratory results, vital signs, and intraoperative information. For laboratory testing variables with multiple measurements, we used only the last preoperative measurements taken within 1 week before surgery for analysis. A total of 118 variables from the electronic database were extracted and listed in Table S1 in Multimedia Appendix 1. Additional extraction details are displayed in supplementary material 1 in Multimedia Appendix 1. The least absolute shrinkage and selection operator (LASSO) method, which could solve high dimensionality and multicollinearity between variables was used. After the initial screening, recursive feature elimination (RFE), combined with 5-fold cross-validation, was adopted to rescreen and select the best hyperparameters [13]. After final screening, missing values were imputed using multiple imputation [14].

### Outcome

The primary end point was the incidence of MINS within the first 30 days after surgery. According to the scientific statement from the American Heart Association [15], MINS was defined as at least 1 postoperative high-sensitivity troponin T of 20 to <65 ng/L with an absolute change ≥5 ng/L or a high-sensitivity troponin T concentration ≥65 ng/L; or at least 1 postoperative measurement of troponin I concentration exceeding the uniform 99th percentile due to a presumed ischemic etiology irrespective of the presence or absence of clinical symptoms and electrocardiographic changes within the first 30 days after noncardiac surgery.

### ML Models

Linear and nonlinear ML models were applied, including category boosting (CatBoost) [16], random forest [17], logistic regression [18], naïve Bayes [19], light gradient boosting machine (LightGBM) [20], extreme gradient boosting (XGBoost) [21], support vector machine [22], and decision tree [23,24]. The above algorithms were implemented using the Scikit-learn, LightGBM, XGBoost, and CatBoost Python packages. Each method is described in detail in supplementary material 2 in Multimedia Appendix 1.

### Model Performance and Evaluation

Because of the imbalance between the positive and negative events, the random under-sampling technique was used to avoid overfitting by the rationale of eliminating samples from the majority class to make the majority class equal to the minority class, which is a simple but effective way to treat imbalanced data sets. Eight ML models with final indicators were developed to predict outcomes. The AUROC was used as the evaluation standard of the model performance, and classifiers with larger AUROCs were considered to have better prediction efficiency, and the best-performing ML model was chosen by its AUROC. We also calculated the 95% CIs for each model using the advanced bootstrap method. Similarly, the related sensitivity, specificity, and accuracy were assessed in models conducted. Appropriate figures were produced for these metrics in the best fitting model, including a precision-recall curve and calibration curve, to show the average precision and difference between the predicted risk and actual risk. The AUROCs were also calculated in the validation sets and in the RCRI model to compare the efficacy.

### Model Interpretation

The SHAP method [24] was used to analyze the importance of features in the model because of the limited interpretability in the ML algorithm. SHAP was used as a scoring metric for feature contributions, through determining the difference between the predicted values with and without each feature for all combinations. The greater the influence a particular value of a sample has on the composition of the model, the farther that point deviates from 0 on the x-axis. Using SHAP values and a summary plot, it is thus possible to determine which features have a significant effect on the prediction and whether this contribution is positive or negative. Moreover, SHAP facilitates individual-level risk prediction and stratification, which is straightforward and understandable by doctors.

### Statistical Analysis

For the baseline data analysis, continuous characteristics were evaluated by the Shapiro-Wilk normality test and analyzed by either the 2-tailed *t* test for normally distributed variables or the Mann-Whitney *U* test for skewed data and were reported as means or medians. Categorical variables were compared using the chi-square test or Fisher exact test and were reported as proportions. As this was a retrospective exploratory study, no attempt was made to estimate the sample size of the study; instead, all eligible patients in the database were included to maximize the statistical power. For all analyses, a 2-sided *P* value <.05 was considered statistically significant. All analyses were performed using Python (version 3.6; Python Software Foundation).

## Results

### Characteristics

**Figure 1.**
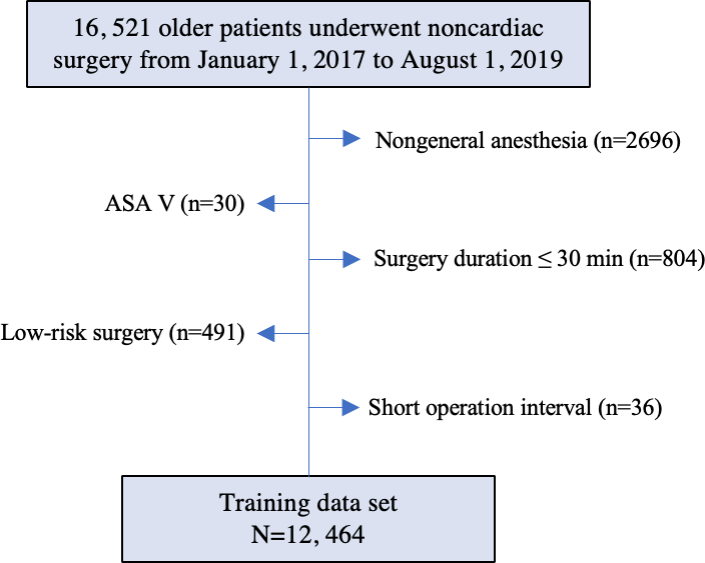
The flowchart of participant selection in the training data set. ASA: American Society of Anesthesiologists.

In total, we retrospectively enrolled 12,464 patients (median age 69, IQR 67-74 years; n= 6793, 54.5% male) who met the inclusion criteria in center 1 from January 2017 to August 2019 as the training data set. Finally, 884 (7.1%) patients developed postoperative 30-day MINS among 12,464 patients. The flowcharts of patient enrollment in the training data set are shown in Figure 1. In the training data set, patients with postoperative MINS tended to be older; have more chronic conditions, such as hypertension, diabetes mellitus, and cerebrovascular diseases; and have more abnormal laboratory test values. The differences in the demographics and other characteristics between patients with and without MINS are summarized in Table 1. The flowcharts of validation data sets are shown in Figure S1 in Multimedia Appendix 1.

**Table 1.** Baseline characteristics of patients with or without myocardial injury after noncardiac surgery (MINS) at center 1 in the training set.

Variable		Non-MINS (n=11,580)	MINS (n=884)	Total (n=12,464)
Age (years), median (IQR)		69 (67-73)	72 (68-78)	69 (67-74)
Hypertension, n (%)		5225 (45.1)	500 (56.6)	5725 (45.9)
Coronary heart disease, n (%)		1282 (11.1)	206 (23.3)	1488 (11.9)
Cerebrovascular disease, n (%)		836 (7.2)	140 (15.8)	976 (7.8)
Renal insufficiency, n (%)		117 (1)	50 (5.7)	167 (1.3)
β-blockers, n (%)		944 (8.2)	140 (15.8)	1084 (8.7)
Diuretics, n (%)		578 (5)	120 (13.6)	698 (5.6)
Anticoagulants, n (%)		845 (7.3)	164 (18.6)	1009 (8.1)
Hemoglobin (g/L), median (IQR)		131 (120-142)	122 (105-136)	131 (120-142)
RBC^a^ (10^9^), median (IQR)		4.32 (3.96-4.65)	4.04 (3.54-4.45)	4.3 (3.94-4.64)
SCr^b^ (umol/L), median (IQR)		71.2 (60.9-82.9)	78.9 (64.8-98.175)	71.6 (61.1-83.7)
RDW^c^ (%), median (IQR)		12.8 (12.3-13.4)	13.3 (12.6-14.4)	12.8 (12.3-13.4)
Albumin (g/L), median (IQR)		40.15 (37.8-42.7)	38.2 (34.7-41.1)	40 (37.6-42.6)
Blood glucose (mmol/L), median (IQR)		5.08 (4.62-5.85)	5.46 (4.77-6.63)	5.1 (4.63-5.9)
Lymphocyte count (10^9^), median (IQR)		0.3 (0.24-0.36)	0.24 (0.18-0.32)	0.3 (0.24-0.36)
Surgery duration (min), median (IQR)		144 (90-205)	180 (120-260)	145 (93-210)
**ASA**^**d**^ **grade, n (%)**				
	I	116 (1)	6 (0.7)	122 (1)
	II	9380 (81)	485 (54.9)	9865 (79.1)
	III	2034 (17.6)	340 (38.5)	2374 (19)
	IV	50 (0.4)	53 (6)	103 (0.8)
Emergency surgery, n (%)		207 (1.8)	76 (8.6)	207 (1.8)
Colloid input (mL), median (IQR)		500 (0-500)	500 (0-1000)	500 (0-500)
Crystalloid input (mL), median (IQR)		1600 (1100-2100)	2000 (1300-2600)	1600 (1100-2100)
Blood loss (mL), median (IQR)		100 (30-200)	150 (50-300)	100 (50-200)
Blood transfusion, n (%)		1044 (9.0)	222 (25.1)	1266 (10.2)
Duration of intraoperative hypotension (min), mean (SD)		16.85 (37.8)	29.91 (57.6)	17.78 (42.3)

a RBC: red blood cell.

b SCr: serum creatinine.

c RDW: red blood cell distribution width.

d ASA: American Society of Anesthesiologists.

### Feature Selection

**Figure 2.**
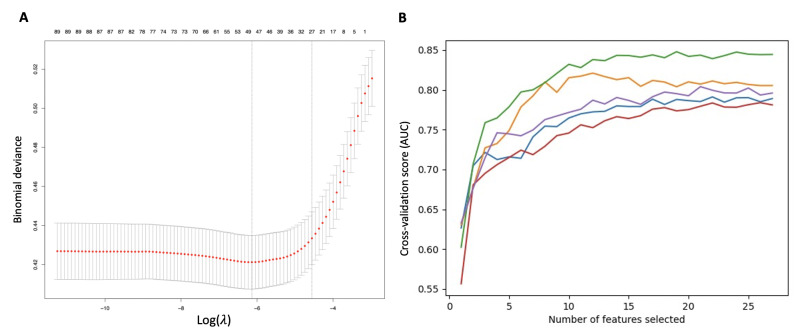
Feature selection by least absolute shrinkage and selection operator (LASSO) and recursive feature elimination (RFE) with 5-fold cross-validation. (A) Through LASSO, the filtered variables were as follows: renal insufficiency, diuretics, cerebrovascular disease, β-blockers, anticoagulants, hypertension, blood transfusion, coronary heart disease, colloid, blood pressure monitoring method, American Society of Anesthesiologists grades, crystalloid, hemoglobin, surgery duration, sodium, age, lymphocyte, anesthesia duration, duration of intraoperative hypotension, red blood cell, glucose, red blood cell distribution width, blood loss, albumin, serum creatinine, facility, and myocardial infarction. (B) Recursive feature elimination with a 5-fold cross-validation method filtered features again and removed 2 parameteres (facility and myocardial infarction), leaving 25 parameters as mentioned above. AUC: area under the curve.

Through LASSO, we found that the optimal number of features for model prediction was 27 (Figure 2A). The RFE method was used to repeat the model building and feature selecting procedure, finally resulting in 25 features by excluding myocardial infarction history and facility (Figure 2B). The features selected by LASSO and RFE are listed in the supplementary material 1 in Multimedia Appendix 1.

### Model Performance and Comparison

**Figure 3.**
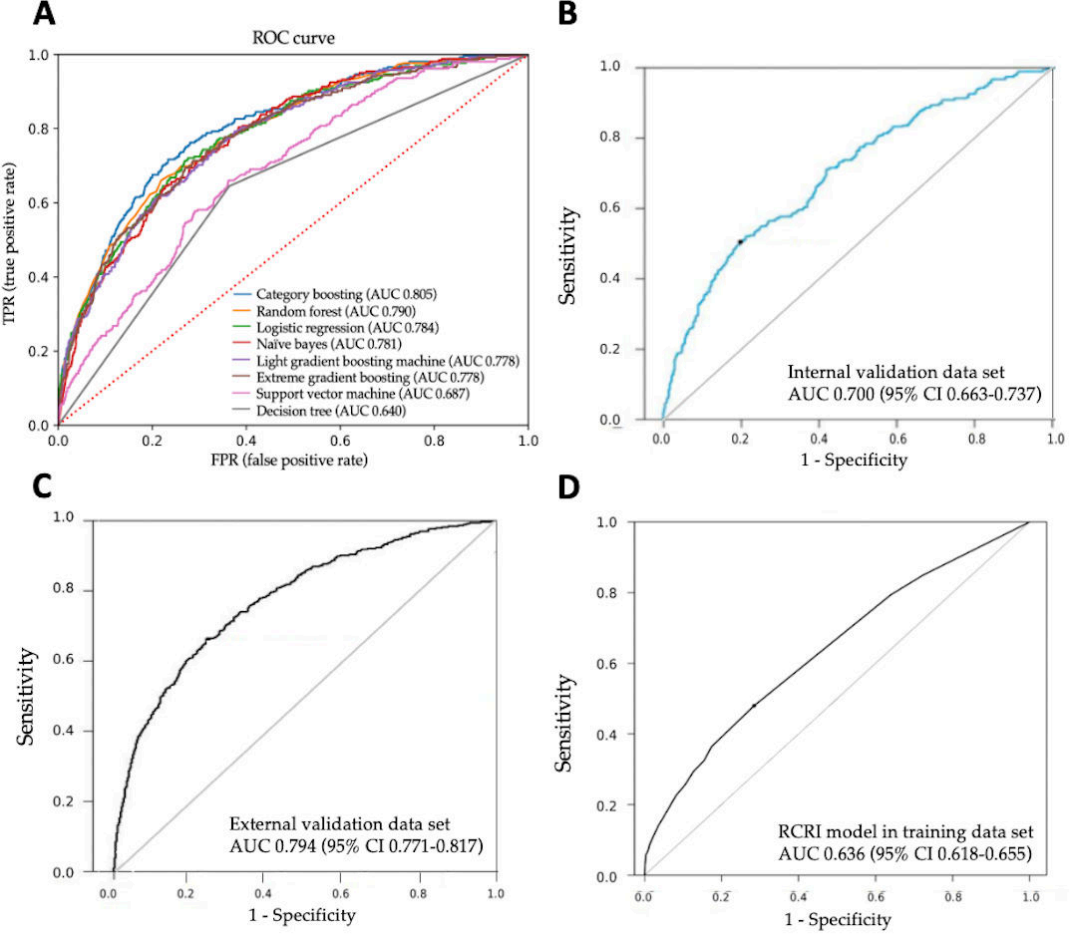
The receiver operating characteristics curve of different models. (A) Eight different machine learning prediction models for myocardial injury after noncardiac surgery using the training data set from center 1. (B) Model performance in the internal validation data set from center 1. (C) Model performance in the external validation data set from center 2. (D) Performance of 6 indicators from Revised Cardiac Risk Index (RCRI) in the training data set. AUC: area under the curve. ROC: receiver operating characteristic.

The training data set from center 1 was used to develop the forecast models, and MINS was predicted with an AUROC of 0.805 (95% CI 0.778‐0.831) by the best-performing CatBoost method, compared with the other 7 algorithms. CatBoost revealed a relatively high accuracy (0.730, 95% CI 0.716‐0.745), sensitivity (0.747, 95% CI 0.694‐0.797), and specificity (0.729, 95% CI 0.714‐0.744). The overall AUROC by all algorithms is shown in Figure 3A. The average accuracy, sensitivity, and specificity calculated by all the algorithms are summarized in Table S2 in Multimedia Appendix 1. The model was well calibrated with a Brier score loss of 0.18; its calibration plot is depicted in Figure S2 in Multimedia Appendix 1.

To verify the stability of our model, prediction was validated with an AUROC of 0.794 in the internal validation set of center 1 and 0.70 in the external validation set of center 2, by the method of CatBoost, and their AUROCs are displayed in the Figure 3B and 3C. Incorporating the 6 parameters in the RCRI model into the validation data set, we observed a poor prediction performance, with an AUROC of 0.636, which was inferior to that of our machine learning models (*P*<.001) (Figure 3D).

### Model Interpretation

**Figure 4.**
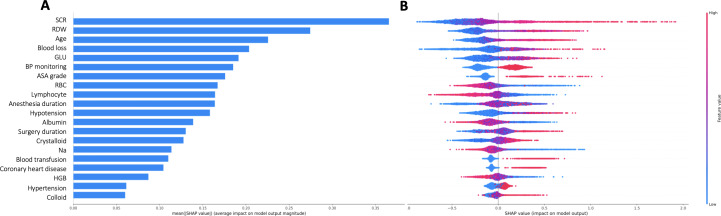
The model’s interpretation. (A) Bar summary of the most important 20 features according to the mean Shapley Additive Explanations (SHAP) values. A higher value of a feature has a greater effect on the model’s composition, indicated by how far a point deviates from 0 on the x-axis. (B) Summary of the most impactful features with interpretation. The red dots represent the high-risk value, and the blue dots represent the low-risk value. ASA: American Society of Anesthesiologists; Na: sodium; HGB: hemoglobin; BP: blood pressure; GLU: glucose; RDW: red blood cell distribution width; SCR: serum creatinine.

Assisted by the development of explainable ML models, the SHAP values for the prediction of MINS were calculated. Figure 4A shows the 20 most influential factors ranked by the average absolute SHAP value, and Figure 4B shows their effect values and interpretations. In the graph, the red dots represent high risk, and the blue dots represent low risk. A higher serum creatinine, higher red blood cell distribution count, older age, increased blood loss, higher blood glucose concentration, higher ASA grade, longer duration of intraoperative hypotension, longer surgery duration, greater infusion of crystalloids or colloids, lower red blood cell count, lower lymphocyte count, lower albumin, lower sodium, and lower hemoglobin were associated with a higher predicted probability of postoperative MINS. Invasive arterial pressure monitoring, blood transfusion, preexisting coronary heart disease, and preexisting hypertension also increased the event risk.

**Figure 5.**
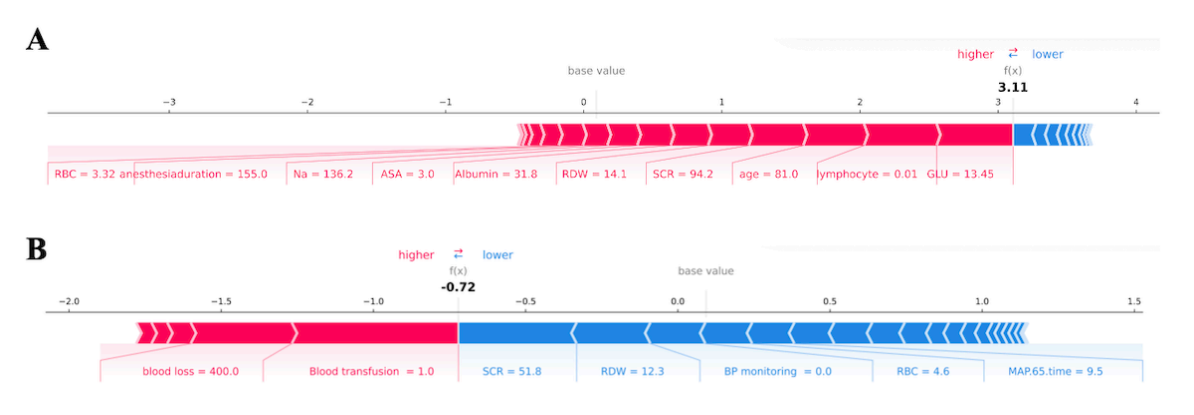
The composition risk of individualized predictions for 2 patients. A blue arrow indicates that a factor reduced the risk of myocardial injury after noncardiac surgery (MINS), whereas a red arrow indicates it increased the risk. (A) An 81-year-old patient with American Society of Anesthesiologists (ASA) grade III underwent surgery with a nearly 2.5-hour duration of anesthesia and developed MINS. (B) A patient with preoperative normal laboratory measurements, with intraoperative blood transfusion, blood loss of 400 mL, and intraoperative short hypotension did not have MINS. ASA: American Society of Anesthesiologists; BP: blood pressure; GLU: glucose; MAP: mean artial pressure; RBC: red blood cell; RDW: red blood cell distribution width; SCR: serum creatinine.

In addition, a visualization method [25] was used to make patient-level prediction interpretations of the model. We provided 2 examples to illustrate this in Figure 5. An 81-year-old patient with ASA grade III underwent surgery with a nearly 2.5-hour duration of anesthesia and developed MINS. His preoperative laboratory test values are listed in Figure 5A. The arrows indicate the influence of each feature on prediction; the red arrows suggest an increased risk of the outcome, and the blue arrows suggest a decreased risk. The predicted score of MINS (approximately 3.11) was 30 times higher than the base value predicted by the model (approximately 0.1). Conversely, the second patient, with preoperative normal laboratory measurements, intraoperative blood transfusion, blood loss of 400 mL, and intraoperative short hypotension, did not experience MINS, with a predicted score of −0.72, lower than the base value of 0.1.

## Discussion

### Principal Findings

In this cohort study, we used ML approaches with multiple demographic and clinical data from EHR to predict the occurrence of postoperative myocardial injury. The CatBoost algorithm achieved the best predictive performance in the training data set and was validated in both the internal and external data sets, with high sensitivity and specificity, also superior to the classic RCRI model. The SHAP method also provided information on the contribution of each variable toward an event or nonevent, quantifying the association between variables and patient outcomes of a single patient. Our results aim to assist in the accurate and timely identification of older patients at high risk of postoperative myocardial injury, enhancing clinical decision support.

The RCRI is considered a conventional predictive model and has been widely used for more than 20 years [6]. Although it has the merit of simplicity with 6 indicators, its use is limited in clinical practice because of its low discriminative ability and lack of specific and sensitive biomarkers for MINS [26]. In our study population, the RCRI model could only achieve an AUROC of 0.636, significantly lower than our model projections. The NSQIP and MICA surgical risk calculators were validated to better estimate cardiovascular risk compared to the RCRI; however, the NSQIP and MICA scores provided only fair discrimination with a C-statistic of 0.70 for postoperative myocardial infarction and MINS outcomes in another external validation research [27]. Our study did not compare our models with the NSQIP surgical risk calculator and MICA as several key indicators needed to be collected prospectively and were not available in our data. Another prediction model by logistic regression was derived from the MANAGE cohort, using only 3 preoperative risk factors, not accounting for intraoperative factors, which might be important contributors to adverse outcomes [9]. Therefore, neither of these widely used assessment tools performed by logistic regression statistics has yet been shown to have sufficient predictive strength and applicability.

Recent work has highlighted the strengths of ML algorithms for predicting postoperative complications compared to classic statistical analyses because they can eliminate nonlinear interactions between clinical variables and resolve the imbalance problem. Oh et al [11] developed the prediction model using extreme gradient boosting algorithm and achieved an AUROC of 0.78 through 12 variables. There were 5 variables coinciding in Oh’s model and ours: operation duration, age, history of chronic kidney disease, history of coronary artery disease, and intraoperative red blood cell transfusion. Other inconsistent variables were due to medication differences, uncollected variables, and number of events. Furthermore, there were 6811 patients selected from 43,019 patients, and the high exclusion rate (84%) and high incidence of MINS (22%) caused a high risk of selection bias in the study. The potential risk factors in Oh’s study may not be generalizable to our data set, which is including older patients. Another ML model was developed by Nolde et al [12], through applying single-layer and multiple-layer variables to different models and achieving the highest AUROC of 0.71. However, the model with optimal prediction efficacy also included information of postoperative vital parameters and oxygenation within 1‐4 days, making it more challenging for anesthesiologists to identify high-risk patients after procedures immediately. Moreover, despite the presentation of variable importance ranking, anesthesiologists and surgeons are were still unable to distinguish modifiable risk factors and make targeted interventions to improve outcomes.

In our study, we used several ML approaches based on different principles and noticed that the prediction efficacy of each approach did not greatly differ from each other, suggesting the promising performance of all advanced ML algorithms for the relatively small and low-dimension data. Logistic regression, representing the simplest of all classifiers, was chosen to create a reference model against the performance of other machine models. Based on this principle, the CatBoost and random forest demonstrated relatively good prediction results in our data set and CatBoost was chosen for further analysis. We also noticed that the naïve Bayes algorithm provided the highest accuracy but with the disadvantage of worse classification performance. The reason for this result might be due to different models dealing with sample classification in different ways. The accuracy index considered only the percentage of correct classification, whereas the AUROC index reflected the ability of a classification model to discriminate between positive and negative samples, taking into account the set threshold’s influence on prediction results. Although similar accuracy can be achieved, the discrimination of being misjudged was not considered while the AUROC index was used as a complementary measure. Based on these points, we conclude that the CatBoost algorithm demonstrates a better predictive effect for MINS due to its highest AUROC, much faster speed, and using default parameters.

In addition, our model not only achieved good predictive effect for MINS but also explored a model-agnostic interpretation technique on how potential variables contribute to adverse outcomes, which was not explored in previous studies. The SHAP values confirmed the importance of variables, reflecting their positive or negative roles. The top important features contributing to adverse cardiovascular complications included preoperative renal dysfunction, inflammatory status, glucose metabolism, anemia, and electrolyte disturbances. The intraoperative hemodynamic and other physiological changes are also important contributors to the occurrence of MINS, including more blood loss, prolonged surgery duration, hypotension, greater infusion of fluids, and blood transfusion [28-34]. The SHAP plot presentats predictions for a single sample in which each feature is a value that increases or decreases the prediction efficacy and its contribution level, providing intuitive explanations for what led to a patient’s predicted risk and quantitative prediction at individual levels. For example, in our first sample patient, we recognized that his high preoperative blood glucose concentration played the greatest negative role in the development of complications. Similarly, in the second sample, intraoperative blood transfusion was considered the strongest risk factor for postoperative MINS. Although the complications are unavoidable mainly due to patients’ comorbidities and surgical stimuli, some variables are modifiable. Identifying specific patient characteristics that predispose them to at-risk status can prompt early targeted prevention or treatments, such as administering insulin to patients with a high blood glucose concentration or taking measures to reduce intraoperative blood loss, which may improve the prognosis. The individual risk estimates may provide the modifiable factors through the SHAP method, which was clinically meaningful and can be used in multiple surgical scenarios.

### Limitations

There are limitations to our study. While the model showed with high accuracy, it was highly dependent on data from EHR. When one indicator was missing, the true risk of adverse outcomes for the patient could not be reflected. Second, the surgical patient data were obtained retrospectively from 2 hospitals, which may have introduced bias, as some potential candidates’ data may not be collected in the EHR. Although external validation was conducted in our model, more validation centers are warranted to support the extrapolation and creditability. Third, some variables were excluded before feature selection, especially those laboratory tests not rountinely measured, such as brain natriuretic peptide and C-reactive protein, leading to omission and neglect of important indicators. Lastly, this study only enrolled older Chinese patients who had noncardiac surgeries from 1 northern center and 1 southern center, and whether the results can be extrapolated to other populations remains uncertain.

### Conclusions

These findings suggest that the ML technique, combining the preoperative and intraoperative variables for predicting MINS with a model-agnostic interpretation, is a potentially efficient management tool for practitioners to guide their postoperative care planning and management.

## Supplementary material

10.2196/54872 Multimedia Appendix 1 Additional information.

## Abbreviations

ASA: American Society of Anesthesiologists
AUROC: area under the receiver operating characteristic
CatBoost: category boosting
EHR: electronic health record
LASSO: least absolute shrinkage and selection operator
MICA: Myocardial Infarction or Cardiac Arrest
MINS: myocardial injury after noncardiac surgery
ML: machine learning
NSQIP: National Surgeons Quality Improvement Program
RCRI: Revised Cardiac Risk Index
RFE: recursive feature elimination
SHAP: Shapley Additive Explanations
TRIPOD: Transparent Reporting of a multivariable prediction model for Individual Prognosis or Diagnosis
