# Supplementary material for: Development and Validation of an Explainable Machine Learning Model for Predicting Myocardial Injury After Noncardiac Surgery in Two Centers in China: Retrospective Study
Source: JMIR Aging. 2024 Jul 26;7:e54872. doi: 10.2196/54872 (PMC11294761; doi:10.2196/54872)

**Supplementary online content**

**Table S1.** List of variables

**Supplementary material 1.** Data extraction steps

**Supplementary material 2.** Model metrics and methods

**Figure S1**. The flow chart of participants selection in validation data sets.

**Table S2.** Details of performance of each model on prediction for MINS.

**Figure S2.** Precision-Recall Curve and Calibration Curve of CatBoost model.

**Table S1. List of variables and data type**

| Number | Variable name | Data type | Number | Variable name | Data type |
| --- | --- | --- | --- | --- | --- |
| **Demographic characteristics** | | | 59 | APTT | Continuous data |
| 1 | Sex | Categorical data | 60 | PT | Continuous data |
| 2 | Age | Continuous | 61 | FIB | Continuous data |
| 3 | Height | Continuous | 62 | INR | Continuous data |
| 4 | Weight | Continuous | 63 | PTA | Continuous data |
| 5 | BMI | Continuous | 64 | D-Dimer | Continuous data |
| **Baseline characteristics** | | | 65 | ALT | Continuous data |
| 6 | Years of education | Continuous | 66 | AST | Continuous data |
| 7 | Marital status | Categorical data | 67 | TB | Continuous data |
| 8 | Functional status | Categorical data | 68 | DBIL | Continuous data |
| 9 | Smoking | Categorical data | 69 | Sodium | Continuous data |
| 10 | Drinking | Categorical data | 70 | Cl | Continuous data |
| **Comorbidities** | | | **Preoperative medications** | | |
| 11 | RCRI high-risk surgery | Categorical data | 71 | NASIDs | Categorical data |
| 12 | Hypertension | Categorical data | 72 | ACEI | Categorical data |
| 13 | Bleeding disorder | Categorical data | 73 | ARB | Categorical data |
| 14 | Abnormal ECG | Categorical data | 74 | Statin | Categorical data |
| 15 | History of surgery | Categorical data | 75 | Bette | Categorical data |
| 16 | Chronic pain | Categorical data | 76 | Chemotherapy | Categorical data |
| 17 | Coronary artery disease | Categorical data | 77 | Diuretic | Categorical data |
| 18 | Arrhythmia | Categorical data | 78 | Anticoagulants | Categorical data |
| 19 | Valvular heart disease | Categorical data | 79 | Antiplatelets | Categorical data |
| 20 | Myocardial infarction | Categorical data | 80 | Beta blockers | Categorical data |
| 21 | Congestive heart failure | Categorical data | 81 | CCB | Categorical data |
| 22 | COPD | Categorical data | 82 | Steroid | Categorical data |
| 23 | Hypothyroidism | Categorical data | **Surgery-related information** | | |
| 24 | Allergic history | Categorical data | 83 | Preoperative SBP | Continuous data |
| 25 | Cancer | Categorical data | 84 | Preoperative DBP | Continuous data |
| 26 | Hepatic insufficiency | Categorical data | 85 | Preoperative MAP | Continuous data |
| 27 | Renal insufficiency | Categorical data | 86 | Preoperative HR | Continuous data |
| 28 | Peripheral artery disease | Categorical data | 87 | Inhalation anesthetics | Categorical data |
| 29 | Cardiac intervention | Categorical data | 88 | Intraoperative anticoagulants | Categorical data |
| **Preoperative lab measurements** | | | 89 | Colloid infusion | Continuous data |
| 30 | Red blood cell count | Continuous data | 90 | Crystal infusion | Continuous data |
| 31 | Platelet count | Continuous data | 91 | Urine output | Continuous data |
| 32 | Hemoglobin | Continuous data | 92 | Blood loss | Continuous data |
| 33 | Serum creatine | Continuous data | 93 | Intraoperative hypothermia | Categorical data |
| 34 | C-reactive protein | Continuous data | 94 | Intraoperative hypoxia | Categorical data |
| 35 | Glucose | Continuous data | 95 | Intraoperative transfusion | Categorical data |
| 36 | Potassium | Continuous data | 96 | Glucocorticoid | Categorical data |
| 37 | NT-pro BNP | Continuous data | 97 | Vasopressor | Categorical data |
| 38 | IDL | Continuous data | 98 | Depressor | Categorical data |
| 39 | LDL | Continuous data | 99 | Blood pressure monitoring | Categorical data |
| 40 | VLDL | Continuous data | 100 | Lowest MAP | Continuous data |
| 41 | HDL | Continuous data | 101 | Intraoperative hypotension | Categorical data |
| 42 | Total triglyceride | Continuous data | 102 | Intraoperative tachycardia | Categorical data |
| 43 | Total cholesterol | Continuous data | 103 | Intraoperative bradycardia | Categorical data |
| 44 | White blood cell count | Continuous data | 104 | Dexamethasone | Categorical data |
| 45 | Neutrophil | Continuous data | 105 | Opioids | Categorical data |
| 46 | Lymphocyte | Continuous data | 106 | Midazolam | Categorical data |
| 47 | Monocyte | Continuous data | 107 | Propofol | Categorical data |
| 48 | Eosinophil | Continuous data | 108 | Sufentanil | Categorical data |
| 49 | Basophil | Continuous data | 109 | Intraoperative NASIDs | Categorical data |
| 50 | MCV | Continuous data | 110 | Surgery duration | Continuous data |
| 51 | MCH | Continuous data | 111 | Anesthesia duration | Continuous data |
| 52 | MCHC | Continuous data | 112 | Anesthesia type | Categorical data |
| 53 | RDW | Continuous data | 113 | PCA | Categorical data |
| 54 | PDW | Continuous data | 114 | Admission to PACU | Categorical data |
| 55 | SII | Continuous data | 115 | Facility | Categorical data |
| 56 | HCT | Continuous data | 116 | Surgery department | Categorical data |
| 57 | Albumin | Continuous data | 117 | ASA grade | Categorical data |
| 58 | TT | Continuous data | 118 | Opioids treatment | Continuous data |

**Supplementary material 1. Data extraction steps**

In this appendix, we describe the process of data extraction that we have accomplished.

1. Extracting from electronic health records, with 118 variables listed in the above table.
2. With criteria to select high-quality features, whose missing rate does not exceed 20%, remaining 92 variables, excluding 26 variables: Years of education, Functional status, Marital status, Bleeding disorder, Abnormal ECG, NT-pro BNP, IDL, LDL, VLDL, HDL, Total triglyceride, Total cholesterol, PTA, TB, DBIL, Sodium, Cl, Preoperative SBP, Preoperative DBP, C-reactive protein, Glucose, Potassium, Chemotherapy, TT, D-Dimer, Eosinophil.
3. Capturing key features through LASSO method from 92 variables, finally with 27 variables: age, facility, preoperative serum creatine, albumin, red blood cell distribution width, blood glucose, red blood cell count, lymphocyte count, sodium, hemoglobin, history of coronary heart disease, hypertension, cerebrovascular disease, renal insufficiency, myocardial infarction, medications of anticoagulants, beta blockers, diuretics, intraoperative blood transfusion, blood loss, invasive blood pressure monitoring, duration of intraoperative hypotension, anesthesia duration, surgery duration, crystalloid and colloid infusion and ASA grade.
4. Estimating the optimal number of features through RFE with five-fold cross-validation, with 25 variables: age, preoperative serum creatine, albumin, red blood cell distribution width, blood glucose, red blood cell count, lymphocyte count, sodium, hemoglobin, history of coronary heart disease, hypertension, cerebrovascular disease, renal insufficiency, medications of anticoagulants, beta blockers, diuretics, intraoperative blood transfusion, blood loss, invasive blood pressure monitoring, duration of intraoperative hypotension, anesthesia duration, surgery duration, crystalloid and colloid infusion and ASA grade.
5. Establishing machine learning model utilizing key features selected above and screening the best algorithm.

**Supplementary material 2. Model metrics and methods**

1. The random undersampling is a classic sampling technique for handling imbalanced data. It can

eliminate samples from the majority class to make the majority class equal to the minority class, which is a simple but effective way to randomly remove the part of the majority class and with robust effect for imbalanced datasets. The method has been validated in some studies and was considered as one of the most mainstream methods.

1. We used the following machine learning methods to train the model, which are the most.

commonly classification method: logistic regression(LR), simple decision tree (DT), random forest (RF), support vector machine (SVM) and light gradient boosting machine (lightGBM), Naïve Bayes (NB) , extreme gradient boosting (XGBoost) and Catboost.

**LR** performs model classification by finding the optimal decision boundary. Each node in the **DT** represents a feature, each link represents a decision rule, and each leaf represents a result. Create a decision tree for the entire data set to get its classification model. **RF** is a classifier that contains multiple decision trees, and its output category is determined by the mode of the output category of individual trees. Compared with a separate decision tree, random forests are less prone to overfitting, and the classification results obtained are more accurate. The basic idea of the **SVM** is to solve the separation hyperplane that can correctly divide the training data set and have the largest geometric interval. Each iteration of **lightGBM** ignores the samples that have been processed well by the previously generated base learner, pays attention to the samples that are difficult to learn by the previous base learner, and trains to get the next base learner. Through steps similar to gradient descent, the base learners are sequentially added until the number of base learners reaches the pre-specified value T, and finally T base learners are combined with weight. **NB** is a simple probabilistic classifier that is based upon Bayes’ theorem and has the assumption of conditional independence of the predictive attributes. **XGBoost** is an optimized distributed gradient boosting library that provides superior prediction through the conversion of a set of weak learners to strong leaners. **CatBoost** is also developed from gradient boosting, following XGBoost and lightGBM, with the strengths of optimized processing of classified features and preventing over-fitting of the models. In addition, CatBoost can capture linear and nonlinear relations between different features and the score of interest, with faster computation and better performance.

**Figure S1. The flow chart of participants selection in validation data sets.**


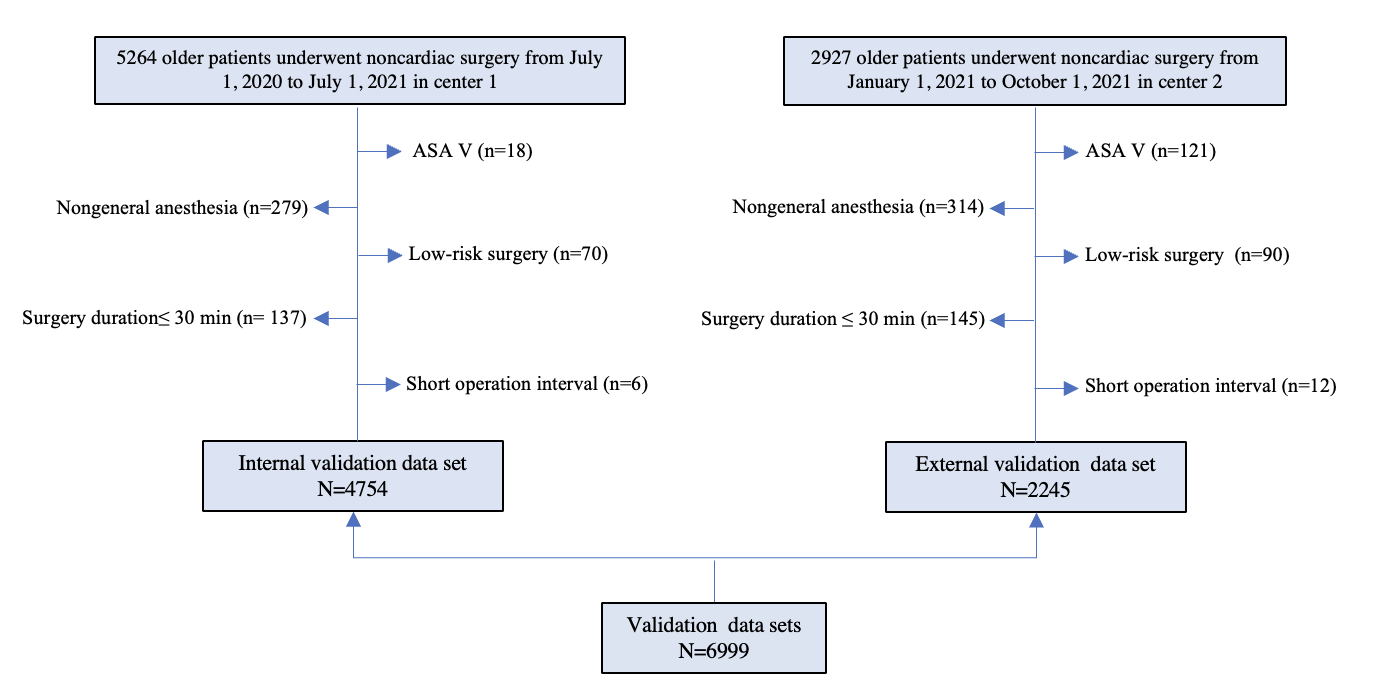


**Table S2. Details of performance of each model on prediction for MINS**

| Model | AUC | Accuracy | Sensitivity | Specificity | F1 score |
| --- | --- | --- | --- | --- | --- |
| CatBoost | 0.805  (0.778-0.831) | 0.730  (0.716-0.745) | 0.747  (0.694-0.797) | 0.729  (0.714-0.744) | 0.2821  (0.2515-0.3129) |
| RF | 0.790  (0.761-0.816) | 0.722  (0.708-0.737) | 0.698  (0.642-0.751) | 0.724  (0.709-0.740) | 0.2628  (0.2320-0.2939) |
| LR | 0.784  (0.755-0.812) | 0.744  (0.730-0.759) | 0.672  (0.614-0.728) | 0.750  (0.735-0.765) | 0.2713  (0.2396-0.3024) |
| XGBoost | 0.778  (0.748-0.807) | 0.714  (0.700-0.729) | 0.706  (0.653-0.762) | 0.715  (0.700-0.731) | 0.2594  (0.2304-0.2910) |
| LightGBM | 0.778  (0.749-0.806) | 0.716  (0.702-0.731) | 0.675  (0.620-0.735) | 0.719  (0.704-0.735) | 0.2523  (0.2231-0.2843) |
| Naive Bayes | 0.781  (0.754-0.807) | 0.801  (0.788-0.814) | 0.532  (0.473-0.593) | 0.822  (0.809-0.834) | 0.2751  (0.2396-0.3105) |
| SVM | 0.687  (0.656-0.718) | 0.626  (0.611-0.642) | 0.660  (0.604-0.716) | 0.624  (0.608-0.640) | 0.2003  (0.1760-0.2261) |
| DT | 0.640  (0.610-0.670) | 0.637  (0.621-0.652) | 0.645  (0.587-0.703) | 0.636  (0.619-0.652) | 0.2009  (0.1756-0.2267) |

**Figure S2. Precision-Recall Curve and Calibration Curve of CatBoost model**


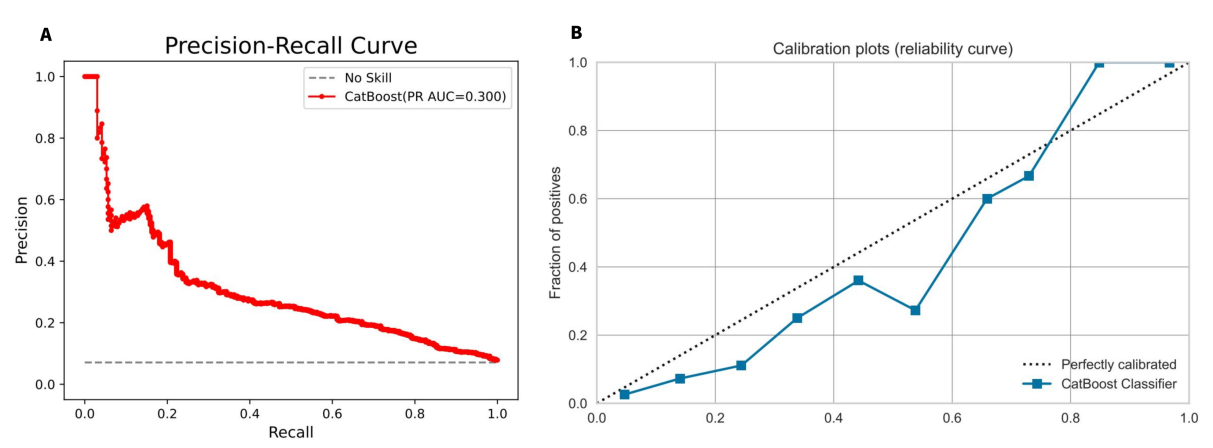

Supplement: Multimedia Appendix 1 [file aging-v7-e54872-s001.docx]
